# Case Report and Literature Review of an Anomalous Course of the Left Main Coronary Artery (LMCA) Arising From the Right Sinus of Valsalva (RSV) Presenting as Takotsubo Cardiomyopathy

**DOI:** 10.7759/cureus.63028

**Published:** 2024-06-24

**Authors:** Sai Rakshith Gaddameedi, Milan Thapa, FNU Arty, Suryansh Atreya, Jayasree Ravilla, Pratik Panchal, Doantrang Du

**Affiliations:** 1 Internal Medicine, Rutgers Health/Monmouth Medical Center, Long Branch, USA; 2 Cardiology, Rutgers Health/Monmouth Medical Center, Long Branch, USA

**Keywords:** coronary artery anomaly (caa), takotsubo-like cardiomyopathy, takotsubo cardiomyopathy, left circumflex artery, left main coronary artery, right sinus of valsalva, left coronary artery anomaly, congenital coronary artery anomaly, embryological coronary artery anomaly

## Abstract

Takotsubo cardiomyopathy (TC) mimics myocardial infarction with symptoms like chest pain, electrocardiogram (EKG) changes, and elevated troponin levels, although it typically features normal coronary arteries upon angiography. While often asymptomatic, coronary artery anomalies (CAAs) can cause intermittent vasospasm and endothelial dysfunction, potentially inducing TC. We report the case of a 74-year-old female with a history of hypertension, hyperlipidemia, and peripheral artery disease, who presented with sudden onset chest pain. Initial EKG and elevated troponin suggested myocardial infarction. However, coronary angiography revealed an anomalous left main coronary artery (LMCA) originating from the right coronary artery (RCA), with no significant stenosis. Subsequent transthoracic echocardiography indicated TC, with the left ventricular ejection fraction improving from 35-40% to 60-65% within days. Cardiac computed tomography angiography (CCTA) revealed that the anomalous LMCA originated from the common trunk at the right sinus of Valsalva (RSV), which further continued as a large, dominant RCA. The LMCA branched into a small to moderate left anterior descending artery (LAD) and a non-dominant left circumflex artery (LCx). The LMCA followed a prepulmonic/anterior course, while the LCx took an interarterial course between the aorta and pulmonary artery. The patient was referred for further surgical evaluation. We conclude that the CAA was an incidental finding and was not related to underlying TC. Although rare, this case suggests a possible correlation between CAAs and a predisposition to stress-induced cardiomyopathy, warranting further investigation.

## Introduction

Takotsubo cardiomyopathy (TC), also known as broken heart syndrome or stress-induced cardiomyopathy, is an acute cardiac condition that occurs due to ballooning of the left ventricle apex with hyperdynamic basal segments in response to a stressor. It can present similar to myocardial infarction with chest pain or pressure, electrocardiogram (EKG) changes including ST-elevation myocardial infarction (STEMI) and elevated troponin. TC can be seen in 1-3% of acute coronary syndrome (ACS) and 0.5-0.9% of STEMI cases [1,2]. It is seen in 80-90% of postmenopausal women above age 50 [3]. Diagnosis is made on coronary angiography, which reveals normal coronary arteries with no occlusion or plaque rupture [4]. TC is usually triggered by any stress or emotional response, which leads to catecholamine surge and subsequent vasoconstriction of coronary vessels. This can result in myocardial stunning and apical ballooning [5]. The steroid hormones released during periods of stress potentiate the action of catecholamines, which can have deleterious effects on cardiac tissues [6]. The anomalous origin of the coronary artery is a congenital defect that is defined by the aberrant defect or origin of three main coronary arteries. It is usually an incidental finding detected on ischemia workup in adults, which has increased its recognition. Coronary artery anomaly (CAA) is usually asymptomatic but can cause intermittent vasospasm and endothelial dysfunction, which can potentially induce TC as it typically affects the apical region of the heart [7]. In one autopsy study, coronary artery anomalies (CAAs) were found to be the second most common cause of sudden death in young athletes [8]. Here, we present a case of TC with an incidental finding of an anomalous left anterior descending artery (LAD).

## Case presentation

A 74-year-old female with a medical history of hypertension, hyperlipidemia, peripheral artery disease, carotid atherosclerosis, mitral valve insufficiency, and left bundle branch block was brought to the hospital for chest pain for one day. On the night before the presentation, she was having dinner with wine when she developed a sudden onset of squeezing chest pain, moderate in intensity, radiating to her back and left arm and associated with shortness of breath. These symptoms gradually improved, but on the next day, she had a recurrence of a similar non-exertional chest discomfort and was brought to the hospital.

The pain resolved with fentanyl, but she still had nausea and vomiting. Her vital signs were within normal limits with a temperature of 98.1 °F, heart Rate of 79 beats per minute, respiratory rate of 16 times per minute, blood pressure of 106/64 mmhg, and saturation at 96% on room air. On physical examination, a 3/6 systolic murmur was heard in the mitral area without radiation, with no other significant findings. Her laboratory work revealed creatinine of 1.25mg/dl, and high-sensitivity troponin was 1.97 ng/mL, with the rest of the complete blood picture and comprehensive metabolic panel unremarkable. Electrocardiogram showed T-wave inversion in the anterolateral (leads I, aVL, V4, V5, and V6) and inferior leads (leads II, III, and aVF).

**Figure 1:**
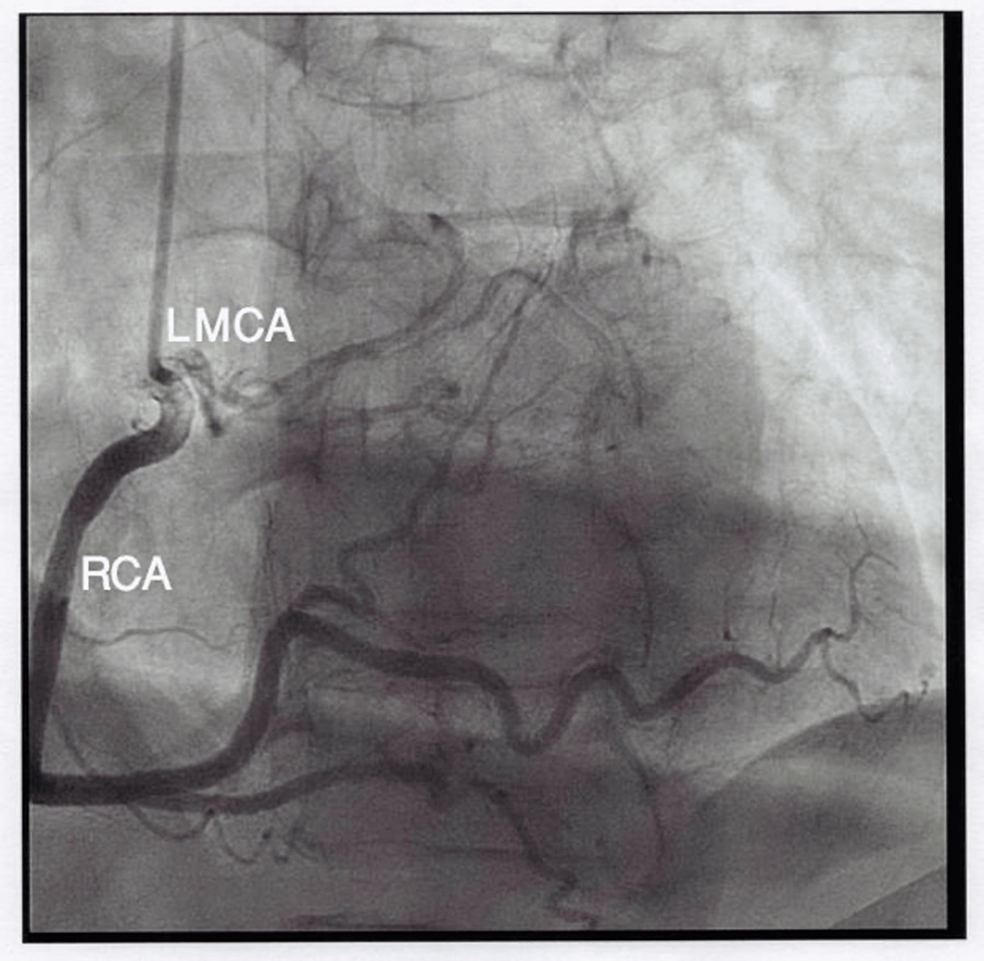
Left heart catheterization images showing the anomalous origin of the left main coronary artery (LMCA) from the right sinus of Valsalva (RSV)

**Figure 2:**
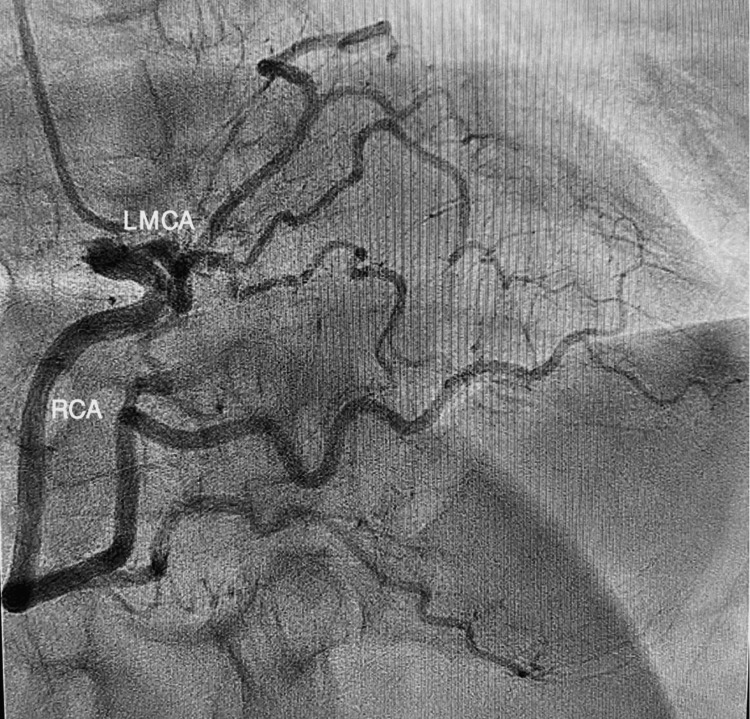
Left heart catheterization image also showing the anomalous origin of the left main coronary artery (LMCA) from the right sinus of Valsalva (RSV)

With a provisional diagnosis and late presentation of myocardial infarction (MI), dual antiplatelet therapy, anticoagulation, and high-dose statin were started. She urgently underwent left heart catheterization (Figures 1, 2), which surprisingly revealed no significant coronary artery stenosis but showed anomalous left main coronary artery (LMCA) originating from the right coronary artery (RCA) with an unclear course of the LAD and left circumflex artery (LCx). It also showed the left ventricular ejection fraction to be 35-40%.

**Figure 3:**
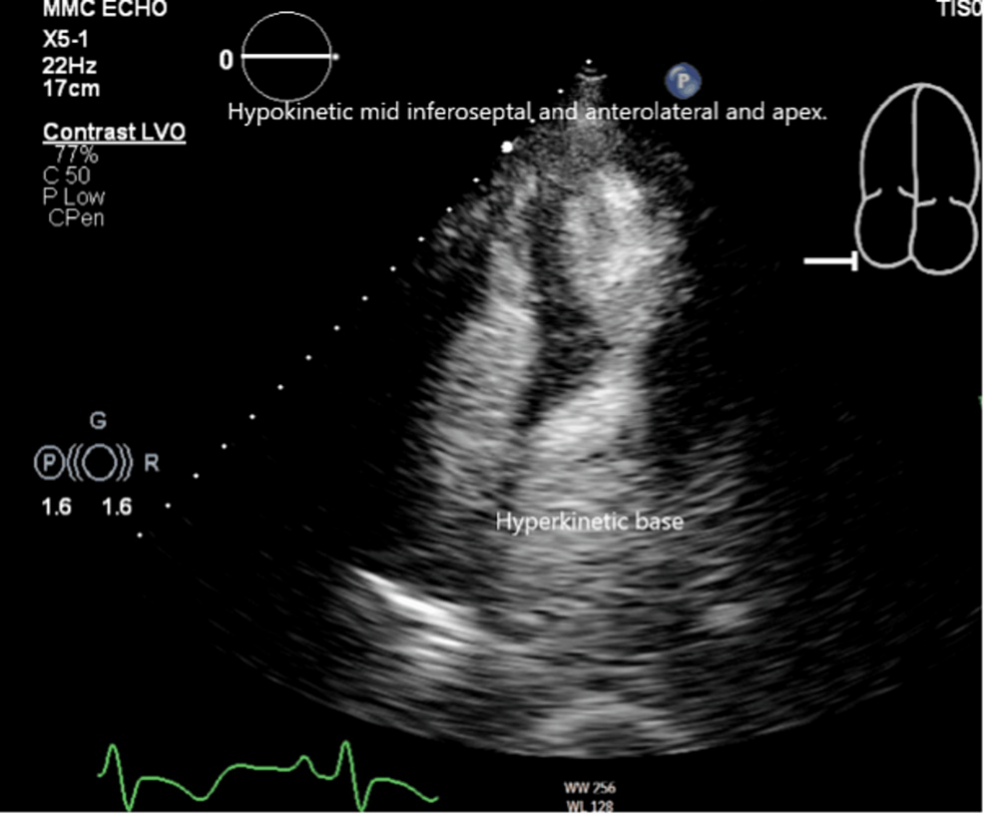
Focal atypical variant of Takotsubo cardiomyopathy

Subsequently, in the following days, transthoracic echocardiography (TTE) (Figure 3) was performed, which revealed mild mid-anteroseptal and apical hypokinesia with basal hyperkinetic segment, suggesting a variant of TC. The ejection fraction normalized to 60-65% with a septal asymmetric left ventricular hypertrophy. She remained chest pain-free and was discharged from the hospital after roughly 25 hours of inpatient management. She was advised to refrain from strenuous exercise until further recommendation, and coronary computed tomography angiography (CCTA) was scheduled in the cardiology office.

**Figure 4:**
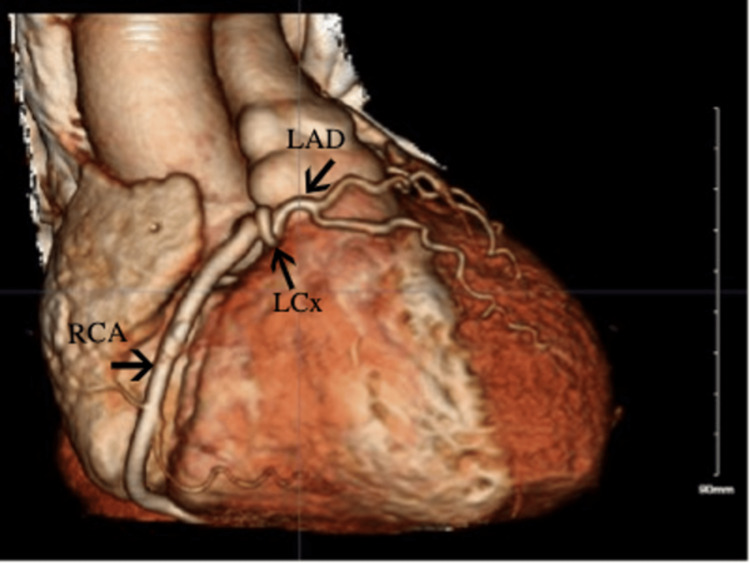
Three-dimensional (3D) image in the coronary computed tomography angiography (CCTA) demonstrating an anomalous coronary artery

**Figure 5:**
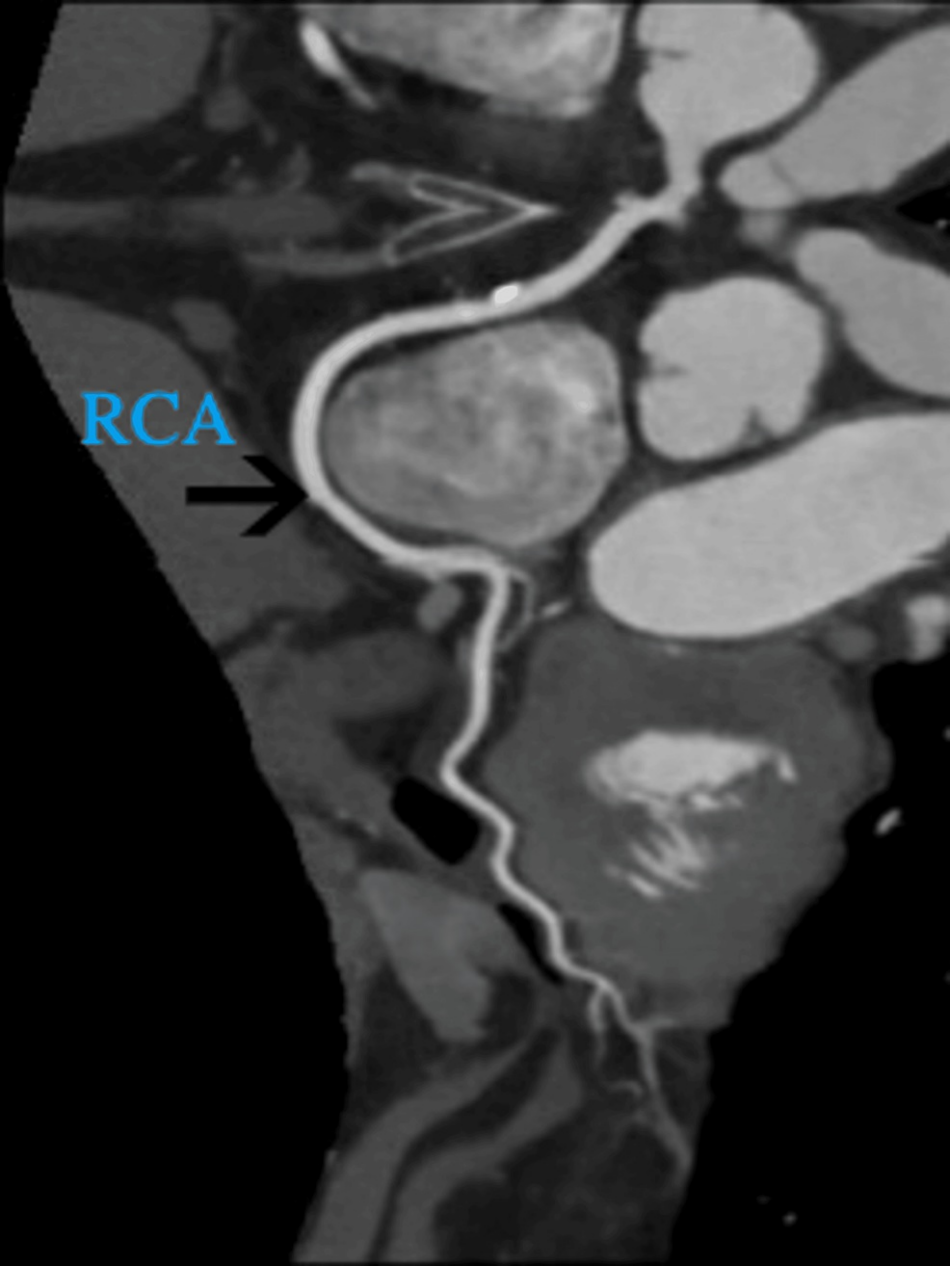
Coronary computed tomography angiography (CCTA) image showing dominant and large right coronary artery (RCA)

**Figure 6:**
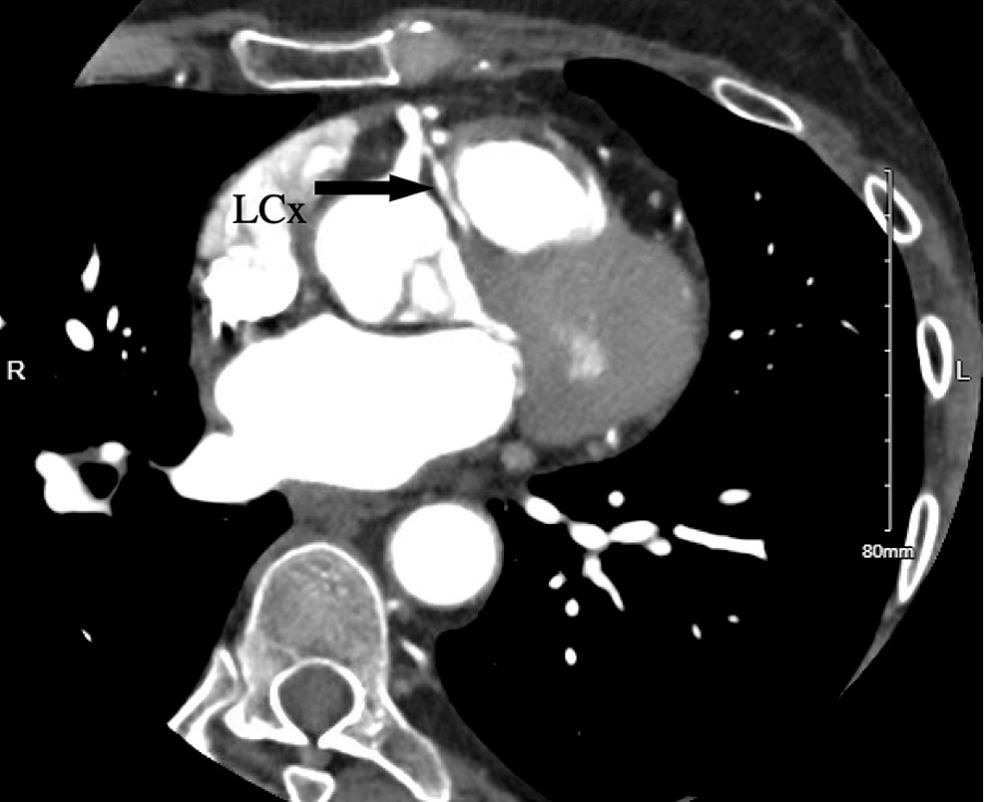
Coronary computed tomography angiography (CCTA) image showing left circumflex artery (LCx) arising from the right sinus of Valsalva (RSV) taking the malignant interarterial course between the pulmonary trunk and ascending aorta

She followed up with a cardiologist after a week and reported a resolution of the chest pain. CCTA (Figure 4) revealed anomalous LMCA arising off the common trunk originating from the right cusp of the sinus of Valsalva (RSV). The common trunk continued further as a large and dominant RCA (Figure 5). The LMCA is divided into small to moderate LAD and non-dominant LCx coronary arteries. The LMCA had a prepulmonic/anterior course while the LCx had an interarterial course between the aorta and pulmonary artery (Figure 6). The EF in the CCTA was 60-65%. Given the benign course of the anomalous coronary artery, she was advised to slowly return to the exercise. She was referred to cardiac surgery outpatient for further management due to concerning anatomical findings of coronary arteries.

## Discussion

Anomalies related to the origin or path of any of the three primary epicardial coronary arteries are referred to as CAAs. These diseases are mostly congenital [9]. CAAs are rare and usually benign conditions that affect a small percentage of the population, ranging from 0.3% to 5.6% [10,11]. They are classified based on the anomalies of origin, course, and termination. Anomalies of origin are subdivided into anomalous pulmonary origin, aortic origin, and congenital atresia of the left main artery. Anomalies of course are subdivided into coronary aneurysm and myocardial bridging. Anomalies of termination include coronary AV fistula and coronary stenosis. These are again subdivided into sub-variants [9]. Our case above is classified under a variant of anomalous aortic origin, which is further classified into a subvariant origin of the LMCA from the right aortic sinus of Valsalva.

According to a study by Yamanaka and Hobbs in 1686, CAAs were found in 126,595 patients who underwent coronary angiography. The study found that 0.017% of the cases involved LCA arising from the RSV, 0.03% involved LAD arising from RSV, and 0.37% involved LCX arising from RSV or RCA [12].

Angina pectoris, chest discomfort, palpitations, dyspnea, dizziness, syncope, and myocardial infarction are among the clinical characteristics linked to CAAs [13]. The presence of CAAs makes cardiac procedures like percutaneous coronary intervention, coronary angiography, or cardiac surgery challenging. Endocarditis, myocardial ischemia, congestive heart failure, and sudden cardiac death are also associated with CAAs [14]. CAAs have been linked to instances of sudden cardiac death (SCD), particularly among young athletes. A study conducted by Maron et al. in 1866, regarded as one of the most comprehensive autopsy examinations to date, found that among athletes who experienced sudden cardiac death, 1,049 cases were attributed to cardiovascular causes. Among these 1,049 cases, CAAs were the second most common cause, accounting for 17% of the total instances [8].

In our case, the patient had an anomalous LCA arising from the RSV. This is considered to be one of the most serious forms of CAAs. A study conducted by Frescura et al. examined 1,200 autopsy specimens and found that 27 of them had isolated anomalous origin of coronary arteries. SCD was seen in 4/4 cases of the LCA origin from the RSV. This indicates a higher risk of SCD in patients with LCA anomaly [15].

The anomalous origin of the LCA from the RSV (LCA-from-RSV anomaly) is further categorized according to the course it takes: the septal route, the anterior free wall route, the retroaortic route, and the interarterial route [16]. In one variant of an anomalous coronary artery originating from the opposite sinus, if the artery takes an interarterial path between the pulmonary artery and the aortic trunk, there is a risk of compression during exertional activities, potentially causing hemodynamic compromise. Nevertheless, an interarterial route does not invariably result in arterial compromise [17]. In our case, the LCA took the benign course of the prepulmonic/anterior route, but the LCx took the malignant interarterial course between the ascending aorta and pulmonary trunk. Due to the potential for compression between the aorta and pulmonary artery and the risk of exercise-induced ischemia, there is a high risk of sudden cardiac death.

For the evaluation of CAAs, initially, coronary angiography was used frequently. However, the current gold standard for studying them is multidetector CCTA due to its non-invasive nature and lower costs. Cardiac magnetic resonance (CMR) is emerging as an alternative to CCTA [13]. According to the American Heart Association (AHA), surgical correction is a grade B recommendation for the anomalous aortic origin of the coronaries from the RSV for symptoms or diagnostic evidence consistent with coronary ischemia attributable to the anomalous coronary artery [9]. Patients with CAAs are at risk for cardiomyopathy due to the sustained ischemic events caused by endothelial dysfunction in the coronary arteries and intermittent vasospasm. In CAAs with an interseptal course, this mechanism can possibly induce TC [7]. However, the apical areas of the myocardium commonly impacted in TC do not align with a particular distribution territory of epicardial coronary vessels; they receive blood supply from various left and right coronary vessels.

We performed a literature review of cases of anomalous coronary arteries presenting with TC and included them in Table 1.

**Table 1:** Review of literature on cases of coronary artery anomalies associated with Takotsubo cardiomyopathy LAD: left anterior descending artery, LCx: left circumflex artery, RCA: right coronary artery, LMCA: left main coronary artery

Author/s	Age and sex	Presenting symptom	Anomalous coronary artery	Type of cardiomyopathy	Management	Outcome	Ejection fraction of the heart
Grani C et al. [18]	74, Female	Respiratory distress	LAD and LCx from dominant RCA	Takotsubo cardiomyopathy	Medical therapy for heart failure	Favorable	20%
González-Jasso J G et al. [19]	76, Female	Chest pain	LMCA coming from the proximal segment of the RCA	Takotsubo cardiomyopathy	Medical therapy	Favorable	45%
Kundapur D et al. [20]	66, Female	Chest pain	RCA from the left coronary sinus and coursing between the aortic root and right ventricular outflow tract.	Takotsubo cardiomyopathy	Surgical correction	Favorable	25-30%
Spina M et al. [21]	66, Female	Chest pain	RCA from the left sinus of Valsalva	Takotsubo-like cardiomyopathy	Medical therapy	Favorable	25%
Liu X et al. [22]	62, Female	Refractory paroxysmal atrial fibrillation	LAD and LCx arising from the RSV	Takotsubo cardiomyopathy	Medical therapy for heart failure	Favorable	50%
Miura S et al. [23]	85, Female	Chest pain	Isolated single L-I type coronary artery anomaly from the left coronary sinus in the absence of right coronary artery	Takotsubo cardiomyopathy	Nonsurgical management	Favorable	N/A

## Conclusions

We conclude that the CAA was an incidental finding that did not require further treatment. We attributed our patient's symptoms to an underlying TC. There are only a handful of cases in literature with coexisting CAAs and TC. Although rare, this case may illustrate a correlation of anomalous coronary anatomy and predilection to stress-induced cardiomyopathy. Further investigation is required to determine a possible causal relationship.
